# Full-Length Genome Sequence of a Novel European Antigenic Variant Strain of Infectious Bursal Disease Virus

**DOI:** 10.1128/mra.00102-22

**Published:** 2022-07-06

**Authors:** Annonciade Molinet, Céline Courtillon, Maryvonne Le Men, Nadia Amenna-Bernard, Charlotte Retaux, Aurélie Leroux, Pierrick Lucas, Yannick Blanchard, Mohammed Nooruzzaman, Mohammad Rafiqul Islam, François-Xavier Briand, Béatrice Grasland, Nicolas Eterradossi, Sébastien Soubies

**Affiliations:** a Agence Nationale de Sécurité Sanitaire de l’Alimentation, de l’Environnement et du Travail, Unité Virologie, Immunité et Parasitologie Aviaires et Cunicoles, OIE Reference Laboratory for Infectious Bursal Disease, Ploufragan, France; b Labocea, Ploufragan, France; c SELAS du Gouessant, Lamballe, France; d Agence Nationale de Sécurité Sanitaire de l’Alimentation, de l’Environnement et du Travail, Unité Génétique Virale et Biosécurité, Ploufragan, France; e Department of Pathology, Faculty of Veterinary Science, Bangladesh Agricultural University, Mymensingh, Bangladesh; DOE Joint Genome Institute

## Abstract

We report the full-length genome sequence (compared to reference sequences) of a novel European variant strain of infectious bursal disease virus (IBDV), designated 19P009381 (AxB1). This should help to further identify such viruses in Europe.

## ANNOUNCEMENT

Infectious bursal disease (IBD) is a ubiquitous immunosuppressive and sometimes lethal disease of young chickens that is induced by IBD virus (IBDV), a member of the *Birnaviridae* family and *Avibirnavirus* genus. IBDV has two double-stranded segments of RNA (1) (A and B). Segment A encodes, among others, the capsid protein VP2. VP2 contains a hypervariable region (HVR), itself containing four loops (2). Segment B encodes the viral polymerase VP1. Viruses from genogroup A2B1 (3, 4) are known as antigenic variants. Sequence information on these strains in Europe is scarce (5, 6).

**FIG 1:**
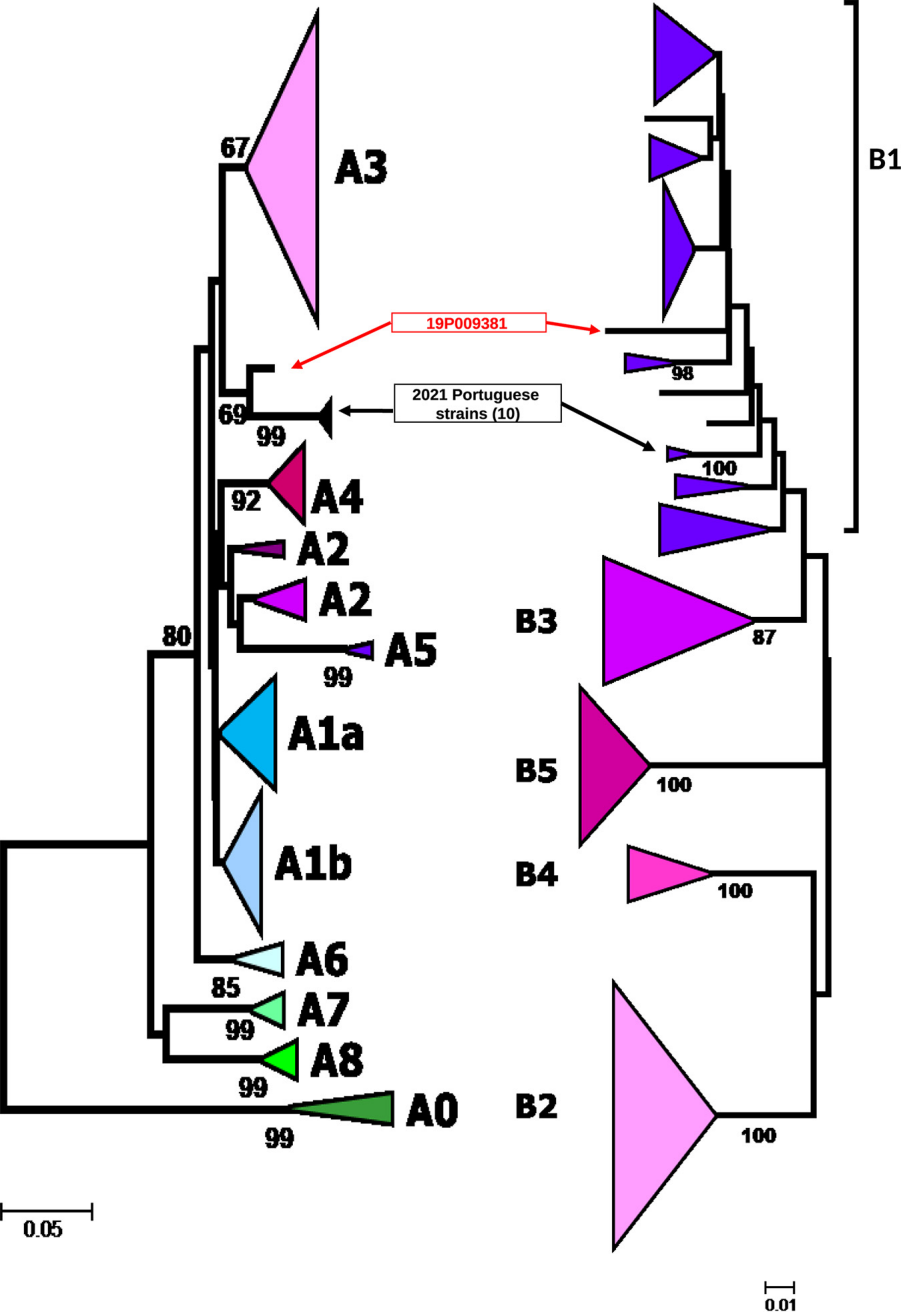
Segment A (partial [355 nucleotides]) and segment B (partial [461 nucleotides]) phylogenetic trees. The evolutionary history was inferred using the neighbor-joining method (9). The percentages of replicate trees in which the associated taxa clustered together in the bootstrap test (1,000 replicates) are shown next to the branches (10). The evolutionary distances were computed using the Kimura two-parameter method (11) and are in the units of the number of base substitutions per site. The analysis involved 492- and 461-nucleotide sequences for segment A and segment B, respectively. All positions containing gaps and missing data were eliminated. There were totals of 355 and 461 positions in the final data sets for segments A and B, respectively. Evolutionary analyses were conducted in MEGA7 (12).

In 2019 in Brittany, France, five bursae of Fabricius (BFs) with lesions evocative of IBDV were pooled for viral RNA extraction using the QIAmp viral RNA kit (Qiagen) and deep sequenced using Ion Proton technology, as described previously, with the Ion Total transcriptome sequencing (high-throughput RNA sequencing) kit (7). A total of 2,387,143 reads, with an average length of 95 nucleotides, were obtained. All tools were run with default parameters unless otherwise specified. Quality assessment was performed with fastqc v0.11.9 and trimming with Trimmomatic v0.36 (options: ILLUMINACLIP=oligo_file.fasta:2:30:5:1:true, LEADING=3, TRAILING=3, MAXINFO=40:0.2, and MINLEN=36). Reference-based assembly using BWA-MEM v0.7.8 and SPAdes v3.10.0 for a *de novo* assembly step and then alignment of the *de novo* consensus sequences to references (GenBank accession number X84034.1 for segment A and GenBank accession number AY459321.1 for segment B) yielded median coverage values of 232× and 154× for segment A (3,261 bp, with a GC content of 53.3%) and segment B (2,827 bp, with a GC content of 53%), respectively. Coverage in the 5′ and 3′ untranslated regions (UTRs) was lower than that in the rest of the genome for both segments (7× and 45× for segment A extremities and 11× and 14× for segment B extremities, respectively), and thus the sequences were obtained by alignment to references. The full-genome consensus sequence (reference number 19P009381) was corrected based on actual reads, using the Integrated Genomic Viewer (IGV) v2.8.10. Nucleotide identity analysis using Molecular Evolutionary Genetics Analysis (MEGA) v7.0 and IBDV sequences available from GenBank used by Islam et al. (4) revealed that the VP2 sequence was close to that of the viral strain variant E (AJ878905, United States, 1985) and to recent Portuguese strains (8) (1/chicken/PRT/7/21 MZ687403 for segment A and MZ687395 for segment B; 1/chicken/PRT/75/21 MZ687404 and MZ687396; 1/chicken/PRT/76/21 MZ687405 and MZ687397; 1/chicken/PRT/118/21 Not retrievable and MZ687398; 1/chicken/PRT/119/21 Not retrievable and MZ687399; 1/chicken/PRT/168/21 MZ687406 and MZ687400; 1/chicken/PRT/189/21 MZ687407 and MZ687401; 1/chicken/PRT/201/21 MZ687408 and MZ687402), with 95% and at least 94% nucleotide identity, respectively. The closest matches for VP1 were strains 9109 (AY459321.1, United States, 2003) and 150127 (MF969108.1, Algeria, 2015), with 97.40% and 97.18% nucleotide identity, respectively. Phylogenetic analyses showed that segment A does not cluster robustly with any described genogroup (Fig. 1), including the A2-US antigenic variant (evolutionary distance of 0.088), although it appears more closely related to a small set of segment A sequences from Portugal. However, segment A of strain 19P009381 diverges from previously defined A lineages by less than 9%. Thus, although it appears to represent genetic elements indicating a new genogroup, more sequences are required for confirmation. Segment B of this virus clustered with genogroup B1 (Fig. 1).

VP2 HVR alignments of strain 19P009381 with classic strains and documented antigenic variants confirmed the presence of mutations associated with altered antigenicity (Table 1).

**TABLE 1:** Mutations associated with altered antigenicity in strain 19P009381

Mutation^*a*^	VP2 loop affected
Pro222Ser	PBC
Gly254Asn	PDE
Gly318Asp	PHI
Asp323Glu	PHI

a Amino acid positions are according to VP2 numbering.

In summary, BLASTn results, amino acid alignments, and phylogenetics support the assertion that French strain 19P009381 is a new example of a European antigenic variant, tentatively assigned to the AxB1 genogroup.

### Data availability.

Raw next-generation sequencing (NGS) reads and full-length and partial sequences for segments A and B were deposited in the SRA and GenBank under accession numbers SRR15808265, OK043826, OK043827, MZ556959, and MZ556960.

## References

[1] Delmas B, Attoui H, Ghosh S, Malik YS, Mundt E, Vakharia VN, ICTV Report Consortium. 2019. ICTV virus taxonomy profile: *Birnaviridae*. J Gen Virol 100:5–6. doi:10.1099/jgv.0.001185. 30484762 PMC12662033

[2] Coulibaly F, Chevalier C, Gutsche I, Pous J, Navaza J, Bressanelli S, Delmas B, Rey FA. 2005. The birnavirus crystal structure reveals structural relationships among icosahedral viruses. Cell 120:761–772. doi:10.1016/j.cell.2005.01.009. 15797378

[3] Michel LO, Jackwood DJ. 2017. Classification of infectious bursal disease virus into genogroups. Arch Virol 162:3661–3670. doi:10.1007/s00705-017-3500-4. 28825213 PMC5671532

[4] Islam MR, Nooruzzaman M, Rahman T, Mumu TT, Rahman MM, Chowdhury EH, Eterradossi N, Muller H. 2021. A unified genotypic classification of infectious bursal disease virus based on both genome segments. Avian Pathol 50:190–206. doi:10.1080/03079457.2021.1873245. 33410703

[5] Jackwood DJ, Cookson KC, Sommer-Wagner SE, Le Galludec H, de Wit JJ. 2006. Molecular characteristics of infectious bursal disease viruses from asymptomatic broiler flocks in Europe. Avian Dis 50:532–536. doi:10.1637/7528-032006R1.1. 17274290

[6] Letzel T, Coulibaly F, Rey FA, Delmas B, Jagt E, van Loon AA, Mundt E. 2007. Molecular and structural bases for the antigenicity of VP2 of infectious bursal disease virus. J Virol 81:12827–12835. doi:10.1128/JVI.01501-07. 17881448 PMC2169122

[7] Abed M, Soubies S, Courtillon C, Briand FX, Allee C, Amelot M, De Boisseson C, Lucas P, Blanchard Y, Belahouel A, Kara R, Essalhi A, Temim S, Khelef D, Eterradossi N. 2018. Infectious bursal disease virus in Algeria: detection of highly pathogenic reassortant viruses. Infect Genet Evol 60:48–57. doi:10.1016/j.meegid.2018.01.029. 29409800

[8] Legnardi M, Franzo G, Tucciarone CM, Koutoulis K, Duarte I, Silva M, Le Tallec B, Cecchinato M. 2021. Detection and molecular characterization of a new genotype of infectious bursal disease virus in Portugal. Avian Pathol 51:97–105. doi:10.1080/03079457.2021.2006606. 34841996

[9] Saitou N, Nei M. 1987. The neighbor-joining method: a new method for reconstructing phylogenetic trees. Mol Biol Evol 4:406–425. doi:10.1093/oxfordjournals.molbev.a040454. 3447015

[10] Felsenstein J. 1985. Confidence limits on phylogenies: an approach using the bootstrap. Evolution 39:783–791. doi:10.1111/j.1558-5646.1985.tb00420.x. 28561359

[11] Kimura M. 1980. A simple method for estimating evolutionary rate of base substitutions through comparative studies of nucleotide sequences. J Mol Evol 16:111–120. doi:10.1007/BF01731581. 7463489

[12] Kumar S, Stecher G, Tamura K. 2016. MEGA7: Molecular Evolutionary Genetics Analysis version 7.0 for bigger datasets. Mol Biol Evol 33:1870–1874. doi:10.1093/molbev/msw054. 27004904 PMC8210823

